# Predicting pediatric optic pathway glioma progression using advanced magnetic resonance image analysis and machine learning

**DOI:** 10.1093/noajnl/vdaa090

**Published:** 2020-08-01

**Authors:** Jared M Pisapia, Hamed Akbari, Martin Rozycki, Jayesh P Thawani, Phillip B Storm, Robert A Avery, Arastoo Vossough, Michael J Fisher, Gregory G Heuer, Christos Davatzikos

**Affiliations:** 1 Department of Neurosurgery, Maria Fareri Children’s Hospital, Westchester Medical Center, Valhalla, New York, USA; 2 Center for Biomedical Image Computing and Analytics, University of Pennsylvania, Philadelphia, Pennsylvania, USA; 3 Division of Neurosurgery, Children’s Hospital of Philadelphia, Philadelphia, Pennsylvania, USA; 4 Department of Neurosurgery, St. Joseph Mercy Health System, Ann Arbor, Michigan, USA; 5 Neuro-Ophthalmology Service, Children’s Hospital of Philadelphia, Philadelphia, Pennsylvania, USA; 6 Division of Neuroradiology, Children’s Hospital of Philadelphia, Philadelphia, Pennsylvania, USA; 7 Division of Oncology, Children’s Hospital of Philadelphia, Philadelphia, Pennsylvania, USA

**Keywords:** diffusion-weighted imaging, machine learning, magnetic resonance imaging, optic pathway glioma, vision decline

## Abstract

**Background:**

Optic pathway gliomas (OPGs) are low-grade tumors of the white matter of the visual system with a highly variable clinical course. The aim of the study was to generate a magnetic resonance imaging (MRI)-based predictive model of OPG tumor progression using advanced image analysis and machine learning techniques.

**Methods:**

We performed a retrospective case–control study of OPG patients managed between 2009 and 2015 at an academic children’s hospital. Progression was defined as radiographic tumor growth or vision decline. To generate the model, optic nerves were manually highlighted and optic radiations (ORs) were segmented using diffusion tractography tools. For each patient, intensity distributions were obtained from within the segmented regions on all imaging sequences, including derivatives of diffusion tensor imaging (DTI). A machine learning algorithm determined the combination of features most predictive of progression.

**Results:**

Nineteen OPG patients with progression were matched to 19 OPG patients without progression. The mean time between most recent follow-up and most recently analyzed MRI was 3.5 ± 1.7 years. Eighty-three MRI studies and 532 extracted features were included. The predictive model achieved an accuracy of 86%, sensitivity of 89%, and specificity of 81%. Fractional anisotropy of the ORs was among the most predictive features (area under the curve 0.83, *P* < 0.05).

**Conclusions:**

Our findings show that image analysis and machine learning can be applied to OPGs to generate a MRI-based predictive model with high accuracy. As OPGs grow along the visual pathway, the most predictive features relate to white matter changes as detected by DTI, especially within ORs.

**Key Points**

- MRI analysis and machine learning may be used to accurately predict OPG progression.
- The model achieved an accuracy of 86%, sensitivity of 89%, and specificity of 81%.
- The most predictive feature relates to white matter changes along optic radiations.

**Importance of the Study**

Although several investigations have sought to identify clinical and radiographic features correlated with the variable clinical course of OPGs, few have made reliable quantitative predictions of tumor behavior over time, making treatment decisions at the time of diagnosis challenging. We successfully apply image analysis and machine learning techniques to predict tumor growth and/or visual acuity decline based on MRI. Multiple features, including changes over time, are used in a computational approach to develop a robust model. The most predictive features relate to white matter changes as detected by DTI, especially within the optic radiations. Such a model may be used to support clinical decision-making in OPG patients related to frequency of surveillance and early initiation of chemotherapy treatment. The findings of the current study set the groundwork for prospective validation in independent cohorts, translation to clinical use, and investigation into predictive models for other low-grade glial tumors.

Optic pathway gliomas (OPGs) are low-grade glial tumors that may occur anywhere along the white matter of the visual pathway, including the optic nerves (ONs), chiasm, tracts, and radiations. They commonly present with decreased visual acuity and may occur in isolation (sporadic OPGs) or in association with Neurofibromatosis 1 (NF1). The incidence of OPGs is significantly higher in patients with NF1. While most children with OPGs have NF1, only 20% of children with NF1 have OPGs.^1,2^ OPGs usually affect children before age 8, although OPGs have been reported in adults as well.^3^ Adult OPGs are uniformly malignant and treated with chemotherapy with or without surgery. In children, however, the natural history of OPGs is highly variable, as tumors may progress, remain stable, or, in some cases, regress over time.^4–6^ For instance, in 1 study of NF1 patients with OPGs and available follow-up data, tumors enlarged in 33%, remained stable in 59%, and spontaneously regressed in 8%.^2^

Given the variable clinical course of OPGs, the initial management strategy commonly consists of surveillance with serial ophthalmologic and magnetic resonance imaging (MRI) examinations.^7–9^ For patients with progressive decline in visual acuity and/or radiographic progression, chemotherapy consisting of carboplatin and vincristine is usually administered as the first-line treatment.^9,10^ In some cases of severe visual impairment, treatment is initiated at the time of diagnosis. Due to the location of OPGs, surgical biopsy or resection is associated with risks of further vision decline, endocrine disturbance, and cerebrovascular injury; it is reserved for tumors with an exophytic or cystic component, usually in the chiasm or hypothalamic region, exerting mass effect or causing hydrocephalus via obstruction of the third ventricle. Surgery may also be offered in cases of severe visual impairment with painful or disfiguring proptosis or instances in which the diagnosis of OPG cannot be made based on imaging characteristics alone.^11,12^ Response to treatment is primarily measured by change in tumor size on MRI and preservation of visual acuity.

Although several investigations have sought to identify clinical and radiographic variables correlated with the clinical course of OPGs, few have made reliable quantitative predictions of tumor behavior over time. OPGs in the setting of NF1 are less likely to be associated with visual impairment at diagnosis and more likely to remain stable over time.^2,13–15^ Also, non-NF1 OPG patients are more likely to have posterior optic pathway involvement, which is associated with a worse prognosis than lesions involving only the ONs and chiasm.^16^ Beyond these clinical observations, features on conventional MRI are poorly associated with vision loss and tumor progression.^2,17^ Other studies using visual-evoked potentials^18^ and positron emission tomography^19^ have failed to show that such modalities predict vision loss. Finally, volumetric studies and optical coherence tomography (OCT) hold potential for providing biomarkers of visual acuity; however, volumetric measurements are limited primarily to the anterior visual pathway, and OCT, which measures structural changes in the retinal nerve fiber layer due to visual pathway injury, requires sedation and specialized equipment for the youngest patients. Both approaches require further prospective evaluation. Thus, the inability to prospectively identify which tumors will progress over time and which will remain indolent makes treatment decisions at the time of diagnosis challenging.^20,21^ We therefore set out to use advanced image analysis and machine learning techniques to generate a predictive model, based on sequential MRI studies, to determine which patients with OPGs will show decline in visual acuity and/or radiographic tumor growth over time. As OPGs are low-grade tumors growing along the white matter pathways of the visual system, we hypothesize that diffusion tensor imaging (DTI), which measures the structural integrity of white mater based on patterns of water diffusion, will yield data predictive of OPG progression.

## Materials and Methods

### Study Design and Patient Population

A retrospective case–control study was performed in order to generate the predictive model. All patients with a diagnosis of OPG confirmed by MRI and managed at a single academic tertiary children’s hospital were identified from an institutional database consisting of data from 2000 to 2015. Patients 2–18 years old at the time of diagnosis were included. Per routine clinical practice, OPG patients underwent surveillance brain MRI every 3 months initially, then extending to every 6 months and then to yearly.^16^ In addition, serial ophthalmological examinations were performed at similar intervals to monitor for decline in visual acuity. All examinations were performed by a neuro-ophthalmologist using age-appropriate testing methods. After age 8, examinations were usually performed annually in patients without prior vision impairment, as new vision decline from OPG is uncommon after this age. Electronic medical records were reviewed to identify patients that showed OPG disease progression, which was defined for the purpose of this study as any enlargement of an OPG on serial MRI as determined by a neuro-radiologist (radiographic progression) and/or any OPG-related decrease in visual acuity as determined by neuro-ophthalmological evaluation. The definition of progression was chosen because vision decline and/or radiographic enlargement are the most common indications for initiation of treatment, such as chemotherapy.^10^ Interval enhancement on T1-contrast enhanced (CE) MRI was not used as an indicator of radiographic progression, as such a finding is not used clinically to guide treatment.^22,23^ Best corrected visual acuity was assessed using age-appropriate testing methods and converted to the logarithm of the minimal angle of resolution (logMAR) to create a linear scale of visual acuity. Worsened visual acuity was defined as a decrease in logMAR of 0.2 or more. Patients at initial presentation with severe OPG-related vision loss, defined as 20/470 or worse were excluded, as per clinical trial guidelines,^24^ as vision could not worsen at subsequent encounters (floor effect). A small subset of patients received chemotherapy at the time of initial presentation and again at a later time after a subsequent progression. These patients and images at the time of progression, rather than the time of diagnosis, were included in the analysis. Patients that underwent surgical biopsy were excluded due to the impact of surgery on the integrity of the white matter tracts.

**Figure 1.**
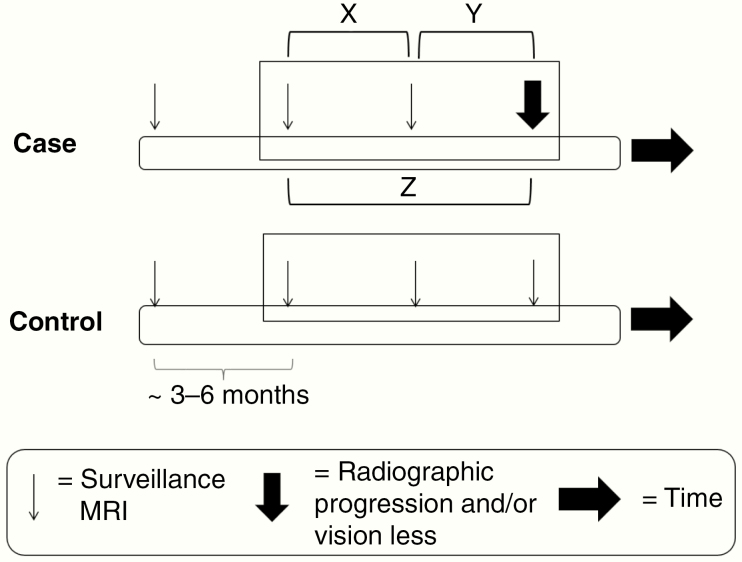
Study schema. Cases and controls were followed by surveillance MRI (represented by multiple downward nonbolded arrows) over time. Among cases, a progression scan is identified (bold downward arrow). The 3 preceding MRIs are included in the analysis (represented by the rectangle) when available. Two preceding MRIs are shown in the diagram for simplicity. For the static study, the 3 studies included in the rectangles are included for analysis. Dynamic study 1 also included changes in variables between scans (represented by brackets X and Y), whereas dynamic study 2 includes changes in variables over time between all combinations of studies (includes bracket Z). MRI, magnetic resonance imaging.

After identifying all patients with radiographic progression and/or visual decline, an equal number of OPG patients were randomly selected from an internal database as control subjects. These patients showed no OPG progression throughout the duration of their follow-up period. For control patients, the 3 most recent consecutive MRI scans were included in the study when available. These MRI studies were termed control scans. Control scans that occurred within no more than 2 years of the most recent control scan were included. For patients with progression, the MRI showing interval enlargement of an OPG and/or the MRI occurring closest in time to the finding of visual decline was termed the progression scan. The 3 consecutive MRI studies preceding the progression scan were collected when available, and these studies were termed the nonprogression scans (Figure 1). Nonprogression scans that occurred within no more than 2 years of the progression scan were included. The study was approved by the Institutional Review Board at the Children’s Hospital of Philadelphia.

### Image Acquisition

Surveillance MRI studies of the brain and orbits were obtained per routine clinical practice, which included the following sequences: T1, T1-CE, T2, T2-fluid-attenuated inversion recovery (FLAIR), and DTI, including the derivatives fractional anisotropy (FA), trace (TR), and radial diffusivity (RAD). All examinations were performed on 3 models of Siemens MRI scanners: Trio (echo time [TE] 91–93 ms, repetition time [TR] 7.3–11.6 s), Skyra (TE 84 ms, TR 9.4–9.6 s), or Verio scanners (TE 91–104 ms, TR 9.4–14 s) (Siemens). Due to requirements for DTI processing, only MRI studies performed on a 3 Tesla magnet were included, and studies performed on 1.5 Tesla magnet were excluded. Inclusion of a DTI sequence as part of the routine protocol for OPG MRI surveillance studies was instituted and optimized for the pediatric population at our institution in late 2008. Thus, studies performed before 2009 were excluded due to incomplete imaging data. Diffusion studies were acquired with an echo planar pulse sequence with 128 × 128 matrix, in-plane voxel size of 2 × 2 mm, and diffusion weighting of *b* = 1000 s/mm^2^. Examinations were obtained with 30 diffusion gradient directions and 2-mm slice thickness.^25^

### Image Analysis and Feature Selection

Multiple imaging features were extracted from each MRI. First, the ONs were manually defined, or segmented, on axial T1-CE sequences from the posterior globe to the anterior half of the optic chiasm (OC) using image analysis software Medical Image Processing, Analysis, and Visualization (MIPAV) version 5.4.4 (mipav.cit.nih.gov). The area of the segmented ONs across all slices was used to compute volume. As OPGs commonly manifest as fusiform enlargements, we collected imaging features related to the morphology of the ONs. Perimeter was computed by calculating the distance between each adjacent pair of voxels around the border of each ON. To compute thickness, a single line was drawn in the center of each ON, and the distance from the central line to the boundary at each voxel was calculated. Tortuosity of the ONs was indirectly measured by calculating the ratio of ON area to perimeter, based on the notion that a more tortuous ON would have a higher perimeter for the same area as compared with a less tortuous ON. For each extracted feature, the minimum, maximum, mean, and standard deviation of the values were calculated and included as additional variables when applicable.

**Figure 2.**
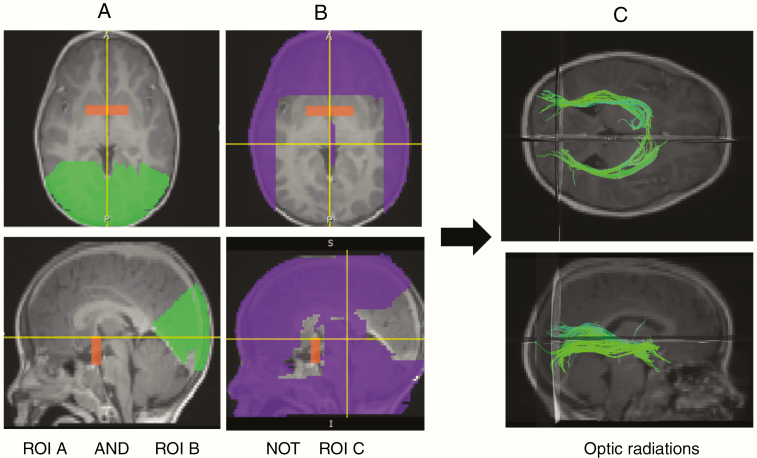
Diffusion tensor imaging of the optic radiations. Using the software Diffusion Toolkit, regions of interest (ROIs) are drawn to include the start (ROI A) and end (ROI B) points of the optic radiations (A). ROI C includes regions not within the occipital lobe (B). By selected tracts between ROA A and ROI B and NOT ROI C, diffusion tensors of the optic radiations are generated (C).

Unlike the ONs, the white matter pathways of the optic radiations (ORs) are not discernible on anatomic imaging. Therefore, DTI was used to define the ORs for each subject. Diffusion tensors were created using Diffusion Toolkit, version 0.6.0.1 (trackvis.org/dtk) and TrackVis (trackvis.org), which are software used to generate, visualize, and analyze fiber track data based on DTI. In Diffusion Toolkit, settings of mask threshold 0.2–1.0 and angle threshold 45 were used to generate the diffusion tensors. To visualize the ORs on TrackVis, multiple regions of interest (ROIs) were drawn on T1 images loaded into TrackVis, such that all tracts running between certain areas and not others were displayed. ROI A was drawn as a large rectangle in the coronal plane that included the posterior portion of the OC and ROI B included all cortex above and below the calcarine fissure and behind the parieto-occipital sulcus. ROI C encompassed all regions not included in ROIs A and B. Tracts were then visualized according to the criteria ROI A AND ROI B NOT ROI C (Figure 2A–C). Using deformable registration,^26^ the 3 ROIs drawn for 1 subject were warped, or transformed, to fit each subsequent subject. Registered ROIs were manually edited when needed. The registered ROIs were applied to each subject to generate tracts of the ORs. The ORs were then segmented using MIPAV for each subject. Regions of tumor extending outside of the ORs as delineated by DTI were not included.

**Figure 3.**
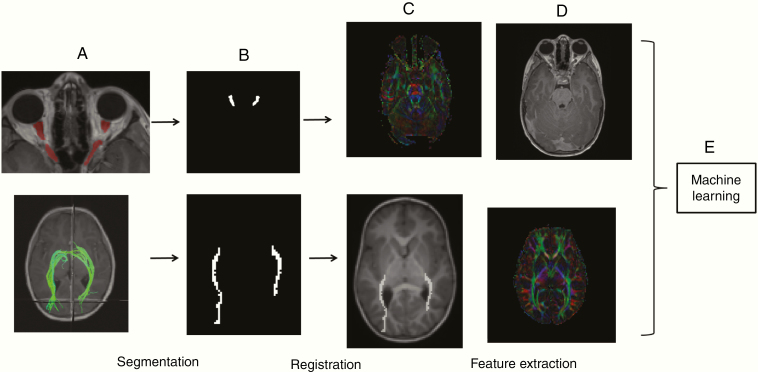
Optic nerve and optic radiation multimodality MRI analysis. After manual defining the optic nerves and generating diffusion tensors for the optic radiations (A), these regions are segmented (B). The segmented regions were then overlaid on all anatomic sequences and DTI sequences, including T1, T1-CE, T2, T2-FLAIR, FA, RAD, and TR through a process of registration (C and D). The distribution of intensities within these regions, as well as minimum, maximum, mean, and standard deviation of values, on all MRI modalities was obtained (feature extraction). These features, or variables, for all patients, served as input for the machine learning algorithm (E). DTI, diffusion tensor imaging; FA, fractional anisotropy; MRI, magnetic resonance imaging; RAD, radial diffusivity; T1-CE, T1-contrast enhanced; T2-FLAIR, T2-fluid-attenuated inversion recovery; TR, trace.

All MRI sequences underwent coregistration, which is a process by which different sets of data are transformed into a single coordinate system. Thus, the regions of the ONs, as defined by manual segmentation, and ORs, as defined by DTI, were identified on the corresponding T1-CE sequences in high-resolution space and low-resolution DTI space (Figure 3A–C). For each patient, intensity distributions, as well as minimum, maximum, mean, and standard deviation of values were determined from within the defined regions across all coregistered imaging sequences, including T1, T1-CE, T2, FLAIR, FA, RAD, and TR (Figure 3C and D). The process was performed for each sequence for each MRI study for each patient. To take into account the varying scale of values, linear histogram matching was performed, after excluding outlier voxels.

### Machine Learning

All extracted MRI-based features served as input for a machine learning algorithm to generate a model predictive of OPG progression (Figure 3E). Support vector machine was chosen as the machine learning algorithm, and data were analyzed using Matlab (MathWorks). Two types of analyses were performed. In the first approach (termed the “static” study), all available MRI studies underwent feature selection, and machine learning was used to find the combination of extracted imaging features that best separated (or classified) the patients as having progression or no progression (a known outcome based on review of the medical record). Features were sequentially added to the model to best predict outcome until no further accuracy was achieved. A final set of features was identified that most accurately classified each patient as having had OPG progression or not. In a second analysis, changes in features over time were included as variables in the model. Patients with more than 1 available imaging study were included. In the first dynamic analysis, changes in variables over time, or between successive imaging studies, were included as additional features in the model. In the second dynamic analysis, changes between all combinations of MRI studies were included. Thus, for a sequence of 3 MRI studies occurring chronologically A, B, then, C, dynamic study 1 included changes in features between A–B and B–C, whereas dynamic study 2 included A–B, B–C, and A–C (Figure 1, brackets).

### Cross-validation and Statistical Analysis

Model performance was evaluated by comparing the classification of each subject by the model (OPG progression or no progression) based on input imaging features, with the known outcome for each patient based on the medical record (OPG progression or no progression). To address overfitting, or the notion that a predictive model will perform exceeding well on the dataset on which it was trained, rather than a new dataset, leave-two-out cross-validation was performed. In this technique, the model was trained on all available subjects except 1 case and 1 control subject. The trained model was then evaluated based on its classification of the 2 excluded subjects. The approach was then repeated for all subjects, excluding a different set of 2 subjects (1 control and 1 case) with each iteration. Accuracy of the predictions was obtained by dividing the sum of true-positive and true-negative findings by the total number of patients. Sensitivity was calculated as the sum of true-positive findings divided by the sum of true-positive and false-negative findings. Specificity was calculated as the number of true-negative findings divided by the sum of true-negative and false-negative findings. A receiver operating characteristics (ROC) analysis was performed, and the area under the curve (AUC) was calculated with a 95% confidence interval. A cutoff point was determined for the optimal sensitivity and specificity, which is the point on the ROC plot closest to (0,1). Baseline characteristics of the patients with and without OPG progression were compared using the Chi-square test and unpaired *t*-test, with the level of significance for a 2-sided comparison set at 5% (*P* < .05).

## Results

Nineteen OPG patients with progression (cases) were matched to 19 OPG patients without progression (controls). Baseline characteristics, including history of NF1, were similar between the 2 groups, except mean age at time of most recent MRI was higher among controls (Table 1). Among cases, 16 patients showed radiographic OPG enlargement with stable visual acuity, 2 patients showed radiographic progression and a decline in visual acuity, and 1 patient had visual decline without radiographic progression. One patient underwent cerebrospinal fluid diversion due to a tectal lesion causing obstructive hydrocephalus; however, the surgery was performed over 1 year after the most recent progression scan. Eleven of 19 OPG patients underwent chemotherapy. Three of the 11 patients received chemotherapy prior to the “progression scan.”

**Table 1.** Baseline Patient and Imaging Characteristics

	Cases	Controls
No.	19	19
Sex (F), No. (%)	7 (37%)	11 (58%)
NF1 status, No. (%)	17 (89%)	19 (100%)
Mean age at most recently analyzed MRI (SD) (years)*	5.1 (2.7)	8.8 (3.2)
Mean time from diagnosis to progression (SD) (years)	2.2 (1.9)	NA
Mean time between most recent follow-up and most recently analyzed MRIs (SD) (years)	NA	3.5 (1.7)
Posterior-most tumor location, No. (%)		
Optic nerves	9 (47%)	11 (58%)
Optic chiasm	5 (26%)	7 (37%)
Optic tract/radiations	5 (26%)	1 (5%)

Cases refer to patients with radiographic progression and/or visual decline, and controls refer to patients without progression during the follow-up period. “Progression” MRI and “nonprogression” MRIs refer to those MRI studies among cases in which the MRI was or was not obtained at the time of progression. F, female; MRI, magnetic resonance imaging; NA, not applicable; NF1, Neurofibromatosis 1; No., number; SD, standard deviation.

*There was no statistically significant difference between all variables, except for a higher mean age at time of most recently analyzed MRI for controls (*P* < .01).

**Figure 4.**
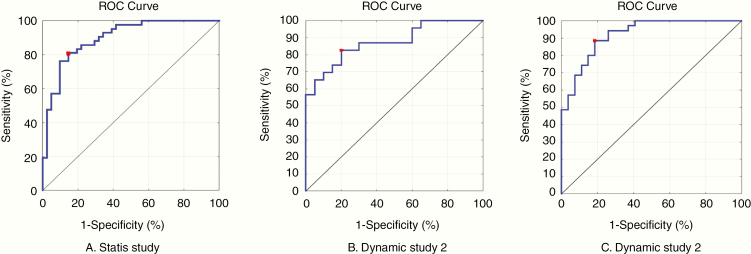
Receiver operating characteristics (ROC) analyses. The ROC curves for the static (A), dynamic 1 (B), and dynamic 2 (C) studies shows sensitivity on the *y*-axis versus 1 − specificity on the *x*-axis. The red dot is the shortest distance from the top left point (0,1) to the ROC curve and represents the optimal threshold. The diagonal is equivalent to chance. Accuracy was determined by leave-two-out cross-validation.

The numbers of time points (MRI studies) for the static and both dynamic studies are shown in Table 2. Of the 19 patients with progression, 16 patients had at least 1 preceding MRI study, and all but 5 patients did not have a progression scan available for analysis. For the static and both dynamic studies, 268 and 532 imaging features were included, respectively. Overall, the most predictive features for progression related to FA and T2 signal within the ORs and included the following: higher intensity of FA values within the OR (AUC 0.83, *P* = 6.7E−07), mean FA values within the OR (AUC 0.80, *P* = 2.6E−05), mean RAD values within the OR (AUC 0.78, *P* = 4.20E−05), and mid-distribution T2 intensity within the ORs (AUC 0.78, *P* = .001). For the most predictive feature, the mean scaled FA values among cases were lower than among controls (6.23 ± 3.56 vs. 2.38 ± 1.79, *P* = 3.3E−07). Among those features that specifically involved the ONs, the most predictive feature was the lower intensity distribution of T2-FLAIR signal, which ranked ninth overall in the model. A list of the 10 most predictive features used in the model are listed in Supplementary Table 1. The highest model accuracy was obtained by the model derived from the dynamic 2 study, with an accuracy of 86%, sensitivity of 89%, and specificity of 81%. Performance metrics of the models found in each study are shown in Table 2 and as ROC curves in Figure 4.

**Table 2.** Performance Metrics of MRI-Based Predictive Models

	Static study	Dynamic study 1	Dynamic study 2
No. MRI studies (time points)	83	43	62
No. of features	268	532	532
Accuracy	83%	82%	86%
AUC	0.88	0.87	0.92
Sensitivity	81%	82%	89%
Specificity	85%	80%	81%

Static study refers to the model created by inclusion of all imaging studies. Dynamic study 1 includes changes in variables over time between sequential scans, and dynamic study 2 includes changes in variables over time between all pairwise combinations of scans. AUC, area under the curve; MRI, magnetic resonance imaging; No., number.

## Discussion

Our findings show that image analysis and machine learning techniques can be applied to OPGs to generate a MRI-based model with high accuracy predictive of OPG radiographic enlargement and/or vision decline over time. Nine of the 10 most predictive imaging features were related to DTI measures, supporting the biological plausibility of the model. Pilocytic astrocytomas, the predominant histological diagnosis in OPGs, grow along white matter of the visual pathways^27,28^ the presence of which is posited to be linked to subsequent visual decline in OPGs.^25^ As DTI detects microstructural changes in white matter, changes in DTI measures are likely to reflect varying degrees of white matter disruption due to tumor involvement. FA, the most predictive factor in our model, describes the degree of directional dependence of water diffusion and is lowest when there is unrestricted diffusion in all directions. With progressive tumor involvement and disruption of water diffusion within white matter tracts, FA is expected to decrease. Correspondingly, FA values were higher in control cases and lower in cases of OPG progression in our study. Similarly, studies of pathologies of white matter pathways of the visual system have shown decreased FA values in disease states, such as multiple sclerosis and optic neuritis, as compared with nondisease states.^29,30^ In the case of OPGs, de Blank et al. showed that FA values of the ORs were associated with visual acuity loss. Furthermore, in a subset of patients, the initial FA values were associated with a trend toward change in visual acuity 1 year later.^25^ Thus, the heavily weighted DTI component of our model supports our initial hypothesis of the importance of this modality in capturing tumor involvement related to the infiltrative nature of OPGs along white matter tracts, which is not apparent on conventional MRI.

The ranking of the features by the machine learning algorithm provided further insight into the significance of the relationship between ORs and tumor progression. The 4 most predictive imaging factors all related to DTI measures within the retrochiasmatic white matter of the visual pathway. Several prior investigations have similarly highlighted the importance of the OR. OPGs involving the posterior visual pathway are more likely to progress than those involving the anterior pathway.^10,16^ Studies of apparent diffusion coefficient (ADC), a diffusion-weighted imaging measure, showed similar values in the anterior optic pathway among patients with and without OPGs, but higher ADC values in the white matter of the OR in OPG patients versus controls.^31^ Also, de Blank et al. found that FA values of the ORs were predictive of visual acuity in OPG patients even when patients with tumors involving the ORs were excluded from analysis.^25^ Other groups have shown changes in OR white matter with tumors limited to the anterior visual pathway.^32,33^ Similarly, in our study, the posterior-most extent of the majority of tumors was the anterior visual pathway, yet the ORs were still among the most predictive features in the model. Technical factors, such as partial volume averaging or susceptibility artifact that may affect DTI measures of the anterior pathway arising from its smaller size compared with the posterior pathways,^34^ or biological reasons, such as subclinical tumor infiltration not recognized on conventional MRI or trans-synaptic anterograde degeneration from disruption of anterior visual input,^35,36^ have been put forth, yet the exact mechanism is unknown. Whether the ORs are involved in the pathophysiology of progression or serve as a biomarker, the top ranking of the ORs in our study is consistent with prior studies and emphasizes the significance of this portion of the visual white matter pathways in progression.

Other groups have investigated the use of features on advanced imaging to predict OPG progression. In a cohort of 12 OPG patients, Yeom et al. showed that ADC was higher on baseline MRI among tumors that required treatment compared with those that did not.^21^ Furthermore, ADC declined after therapy in several cases. Although this study supports the use of diffusion-weighted imaging in predicting OPG behavior, the investigators focused on chiasm tumors only, whereas our model applied features extracted from the entire optic pathway, including the ORs. Jittapiromsak et al. utilized dynamic contrast-enhanced (DCE) MRI, which reflects the microcirculatory structure and vascular permeability of tissues, to show that derived measures were significantly higher among progressive OPGs, achieving sensitivity of 100%, specificity of 93%, and accuracy of 95%.^37^ However, the study involved a small sample size that included 5 out of 20 total patients with progression, and the high performance of the model may be due to overfitting, as cross-validation was not performed. In the same study, ON features such as dural ectasia, tortuosity, and perineural thickening were not predictive of progression. Similarly, the most predictive features related to the anterior white matter pathway in our model, T2-FLAIR signal of the ON, ranked ninth in our model. Our group has previously reported that ON tortuosity does not predispose to aggressive OPG with associated vision loss.^38^ Finally, in a study of both DCE and diffusion-weighted imaging, Jost et al. found clinically aggressive OPGs had significantly higher mean DCE permeability values compared with stable tumors and no difference between mean diffusivity values found on diffusion-weighted imaging.^17^ Although the latter findings differ from our study findings, half of the subjects in the study by Jost et al. had prior treatment, which may have influenced the white matter integrity separate from effects of tumor progression. In our study, all imaging data used to build the model was prior to receiving recent chemotherapy. DCE was not available as part of routine imaging in our patient population and therefore not included in the current study.

Our study was designed taking into account the strategy of obtaining serial MRIs when managing OPG patients and has several clinical applications. Whereas prior investigations compare MRI studies between patients with tumors that have progressed versus those with tumors that have not, we include the progression MRI as well as preceding scans among patients with progression versus several MRIs in control patients. We are most interested in the imaging features leading up to progression, rather than only the features that define clinical progression itself, although both sets of features (preprogression and progression) are included in our model. In addition, we include as variables within the analysis the difference between multiple MRI features over time. The improved accuracy when comparing the dynamic 2 study versus the static and dynamic 1 studies demonstrates the added benefit of including change over time in building a more robust model. This observation is similar to clinical practice in which differences in tumor growth may appear minimal when comparing 2 recent surveillance scans but become more apparent when comparing the most recent scan to more distant, earlier imaging studies. Due to the difficulty in predicting OPG progression, children may have significant vision loss by the time progression is diagnosed and before treatment is initiated. Thus, patients identified as being at high risk of progression could undergo more frequent surveillance such that progression may be detected earlier than with routine surveillance. Earlier initiation of treatment, in turn, may result in improved tumor control rates and decreased loss of vision. Furthermore, the ophthalmologic examination is difficult in young children, especially in NF1 patients who may have an associated disorders of attention.^39^ In cases where ophthalmological examination is inconclusive or discordant from radiographic studies, the predictive model can provide objective data to support the decision of whether or not to initiate treatment.^21^

Despite the strengths of the computational approach applied and clinical relevance, our study has several limitations. First, data were collected retrospectively. Cohorts matched for age, sex, and tumor location are preferred; however, the patient sample was limited to available MRI scans on a 3 Tesla magnet after 2009 when DTI was routinely performed for all OPG patients. For this reason, the definition of progression was purposefully broadened and not limited to a defined percentage of tumor growth in order to increase our sample size and provide the highest possible number of inputs into the machine learning algorithm. In addition, multiple imaging studies in time were included for each patient when available. Also, age and gender were not found to be predictive of progression in the model. Second, it was not possible to ensure that all control subjects did not progress after time of most recent follow-up; however, the mean age at most recent follow-up for controls was 8.8, and OPG patients are unlikely to have vision decline after this age. Third, the majority of patients in the study had NF1, and there are significant differences between tumors in NF1 and non-NF1 patients.^2,40^ While the number of patients with and without NF1 was similar in both groups, the results may not generalize similar to non-NF1 patients. Fourth, despite performing cross-validation, the gold standard for testing the performance of a model derived by machine learning is to run the model on a separate dataset. The incorporation of multiple types of imaging features helps raise the likelihood of generalizability of our model, whereas studies focusing on a single parameter, such as FA values, may be subject to overfitting and perform less well when applied to a novel dataset. Nonetheless, in future studies, prospective investigation of an independent OPG cohort with defined tumor progression and vision decline would address such limitations. Although deformable registration was used to decrease any inconsistencies in ROIs between patients when creating DTI tracts, to increase the likelihood of clinical translation, new automated techniques for tumor segmentation and creation of DTI tracts may be applied to standardize the approach and decrease the required computational time.^41–43^

## Conclusion

Image analysis and machine learning can be applied to OPGs to generate a predictive model of progression (radiographic enlargement and/or vision decline) with high accuracy. The model incorporated multiple imaging features and used a computational approach. As OPGs are intimately associated with the visual pathway, the most predictive features relate to white matter changes as detected by DTI, especially within the ORs. Incorporating the change in features over time increased the accuracy of the predictive model. Such a model may be used to support clinical decision-making related to frequency of surveillance and early initiation of treatment. Future studies will focus on prospective validation in independent cohorts and take advantage of new software for feature extraction and/or application to other low-grade glial tumors.

## Supplementary Material

vdaa090_suppl_Supplementary_Table_1

## Notes

**Authorship Statement**. Conception and design: J.M.P., M.J.F., G.G.H., and J.P.T; acquisition of data: J.M.P., H.A., M.R., A.V., and R.A.A.; analysis and interpretation of data: H.A. and J.M.P.; writing of the manuscript: J.M.P.; critical review of the manuscript: J.M.P, H.A., M.J.F., A.V., R.A.A, P.B.S., G.G.H., and C.D.; study supervisors: G.G.H. and C.D.
