# Supplementary material for: Predicting pediatric optic pathway glioma progression using advanced magnetic resonance image analysis and machine learning
Source: Neurooncol Adv. 2020 Aug 1;2(1):vdaa090. doi: 10.1093/noajnl/vdaa090 (PMC7455885; doi:10.1093/noajnl/vdaa090)
Supplement: vdaa090_suppl_Supplementary_Table_1 [file vdaa090_suppl_supplementary_table_1.docx]

**Table S1.** Most predictive features of the predictive model listed in descending order.

| **Ranking** | **Feature** | **AUC** |
| --- | --- | --- |
| 1 | FA value of OR (bin 8) | 0.83 |
| 2 | FA value of OR (bin 7) | 0.80 |
| 3 | Mean FA values of OR | 0.80 |
| 4 | Mean RAD of OR | 0.78 |
| 5 | STD of FA values of OR | 0.78 |
| 6 | T2 value of OR | 0.78 |
| 7 | Mean T2 values of OR | 0.78 |
| 8 | T2 value of OR (bin 6) | 0.78 |
| 9 | FLAIR value of ON (bin 1) | 0.77 |
| 10 | T2 value of OR (bin 2) | 0.76 |

Abbreviations: AUC = area under the curve, FA = fractional anisotropy, FLAIR = fluid-attenuated inversion recovery, ON = optic nerves, OR = optic radiations, RAD = radial diffusivity, STD = standard deviation. Bins refer to ranges of values used for a given variable used for converting a variable from continuous to categorical for the purpose of normalization and analysis.
